# How do Internet moms raise children? The reshaping of Chinese urban women’s parenting psychology by COVID-19 online practices

**DOI:** 10.3389/fpsyg.2022.933582

**Published:** 2022-08-10

**Authors:** Ru Zhao, Gaofei Ju

**Affiliations:** ^1^School of Journalism and Communication, Northwest University, Xi’an, China; ^2^School of Journalism and Information Communication, Huazhong University of Science and Technology, Wuhan, China

**Keywords:** “Internet moms”’ parenting psychology, parenting practices, urban women, mediatization, parenting psychology

## Abstract

With the acceleration of social transformation and “mediatization,” urban women’s parenting practices have become an important factor affecting the demographic structure and national development. The global COVID-19 pandemic has further contributed to the networking of social life and the creation of “Internet moms” who rely on the Internet for parenting interactions. Using a mixed-methods design, this paper conducted participant observation and in-depth interviews with 90 mothers from various industries born after 1980/1990 across multiple geographies in China to examine the impact of urban women’s Internet practices on the psychology and practice of parenting during the COVID-19 pandemic, as well as how they were empowered by media technologies to practice motherhood and complete their role socialization through the sharing of parenting information, experiences, and actions. The purpose of this qualitative study was to investigate the changing impact of Internet-based parenting practices on Chinese urban women’s daily lives during the COVID-19 pandemic. Through the analysis of these influences, it was found that the whole society, including urban parenting groups, paid attention to self-expression and self-worth and further hoped to arouse society’s recognition, face up to the identity of “mother” and “female,” and give more attention and support to women. The study also found that, as interpersonal communication channels were hindered during the COVID-19 pandemic, the power of the Internet, represented by social media, has created a new platform for information empowerment, action mutual, and ideation of motherhood for urban women formerly bound to family and parenting matters. From individual, family, and individual parenting experiences to group, social, and shared scenarios, urban women are engaged in emotional and memory interactions, including motherhood-related expression, experiences, and collaboration. This shift from virtual to physical has reshaped their parenting view, helping them break through the confines of family experience and traditional customs in addition to providing psychological motivation to express their gender concepts, shape their self-image, construct gender power, and interpret intimate relationships, pushing them to become more reflective of the times, as well as more capable and authoritative.

## Introduction

Raising children has long been the focus of family activities. In general, mothers have been the main performers of this child-rearing work. There are rich social factors behind what seems to be a natural ability among ordinary women. Thousands of years of civilization in China have left behind not only a rich historical and cultural heritage but also deep and complex social customs and social experiences. In traditional agricultural societies, parenting experience tends to rely on intergenerational inheritance for various reasons, including social and family structures. These appear in child-rearing as a set of mature experience system constraints from preparation for pregnancy to child-rearing. Women tend to be the primary bearers of these constraints. With the interaction of multiple factors including technological advances, improvements in health care, and social changes, traditional parenting practices, and experiences are gradually being abandoned. Women entering urban life are reluctant to trust traditional parenting experiences because they have different parenting concepts (Baranowski et al., 2021), while lacking access to traditional parenting knowledge. These women need to create a whole new knowledge system around child-rearing, from pregnancy, birth to parenting, and education. Modern society’s neglect of the topic of women’s childcare has also led women to take on more and more social responsibilities with less and less social support (Van Cleaf, 2018). As a result, the transition to motherhood has led to a complex and socially unsupportive “social disconnect” (Britton et al., 2019), and urban women are often caught up in contemplating “How do I parent effectively?” (Carlson, 2017), and “how to parent scientifically.”

As the new media power of multiple technologies penetrates daily life, human society has gradually moved closer toward “mediatization” (Hepp et al., 2015). The “mediatized life” has become an important part of people’s social life. How mediatized societies reconfigure our social world (Mazumdar et al., 2021), how journalism and public attention are constructed (Kunelius and Reunanen, 2016), and how people are responding to these changes (Nadia et al., 2019) have become central keywords in exploring the interrelationships between people and the media, technology, and even other people, as well as between media and media. Mediatization has changed the traditional mode of information production and consumption and has extended the boundaries of content production and communication to the general public. Today’s digital devices have become an important part of the human body, and the development of the Internet has made it deeply embedded in people’s daily lives, making the media a true “extension of people” through the deep link between the virtual and the real. State institutions, non-governmental organizations, social groups, and individuals, all are included in the digital media environment. In this context, urban women are also using these media in new ways to access a wealth of information about parenting. In this social environment, urban women’s parenting practices are naturally embedded in a mediatized society that is throwing off the shackles of family and parenting matters through the Internet and bridging the gap between traditional and scientific parenting (Zhu et al., 2019). Urban mothers are beginning to explore new virtual social interactions based on motherhood. Through virtual community interactions, urban mothers are writing and expressing themselves online as part of digital identity, shaping their self-image, constructing gender power, and interpreting intimate relationships. They are learning about scientific parenting, exchanging parenting experiences, and sharing parenting stories thus creating an “online parenting” (Florean et al., 2020) knowledge system that differs from traditional parenting concepts. As a result, their parenting psychology has also changed. The framework of mediatization provides a new theoretical perspective to interpret current cultural and social changes (Hjarvard, 2014). Considering urban women’s mediatized parenting practices from this perspective is thus relevant for analyzing the mediatized survival and expression of the socially disadvantaged group of urban women raising children.

The global COVID-19 pandemic has increased the role of the Internet in daily life practices, promoting the process of mediatization across society as a whole and influencing the way people think and practice. According to the 49th Statistical Report on the Development of the Internet in China released by the China Internet Network Information Center (CNNIC) (2022), as of December 2021, China’s Internet users spent 28.5 h online per capita per week, up 2.3 h from December 2020, and the Internet has been deeply integrated into people’s daily lives. Managing the parent–child and family relationships during the pandemic is not only a family issue but also a social issue. There are numerous new perspectives for analyzing family social relations during the pandemic, which have included focusing on parenting stress in young parents (Adams et al., 2021), parenting styles and parent–child relationships (Chung et al., 2020), differences in parenting attitudes (Forbes et al., 2021), parental psychological flexibility (Gould et al., 2020), and the relationship between parenting and parental burnout (Bastiaansen et al., 2021). For the underprivileged group of urban mothers (Mazumdar et al., 2021), the COVID-19 pandemic has further detached their child-rearing practices from their daily life and derailed their socialization process. The pandemic has also prompted them to write new stories in the new Internet era. According to the “China Parenting Report under COVID-19” released by China’s professional parenting platform Parenting Yuer.com (Yuer, 2020), the daily activity of parenting online communities increased by 22% year-on-year during COVID-19, and the daily activity of online consultation services increased by 50.58%. Urban mothers seek and establish social relationships through social media to overcome the lack of social interaction in parenting practices during the COVID-19 pandemic via para-social interaction. Although mothers tend to be primarily responsible for parenting, the parenting practices and psychological changes in this underprivileged group during the pandemic have failed to receive widespread attention. In this context, urban women who are raising children have to cope with the impact of the “social isolation” of the COVID-19 pandemic on their parenting activities, while also trying to adapt to the interaction between media and COVID-19 pandemic in answering the question, “How should I raise my children?” With the influence of COVID-19 on everyday social life, more and more young mothers are choosing online parenting as a new way of parenting. This shift in parenting is both a change in parenting behavior and the result of mediated and social interaction. Today, mediated parenting is becoming a new and more popular form of parenting, reflecting not only the mediated nature of everyday parenting among Chinese urban parenting women but also the daily life of mediatized parenting. The impact of the COVID-19 pandemic on the health of women in urban areas is not only related to changes in parenting styles and attitudes but also has a profound impact on family structures, social structures, and the education of young people. Considering this group as the entry point for research, we can not only analyze the impact of the COVID-19 pandemic on the parenting activities of urban women in the context of the interaction between media and socialization and further provide a new perspective for exploring the impact of the COVID-19 pandemic on Chinese families and society, but also draw the attention of the whole society, including urban parenting groups, to self-expression and self-worth through the discussion of motherhood. From this perspective, the study of the impact of the COVID-19 pandemic on the parenting psychology and parenting activities of urban women with children has both practical urgency and social value. It has been argued that media becomes powerful only when it is integrated with practice, unleashing its “molding force” (Hepp, 2012). To respond to the concerns of this group, this study therefore uses the mediatized society as a theoretical framework and investigates the mediated parenting practices of Chinese urban women during the COVID-19 pandemic in depth, as well as the reshaping of their cognition and behavior by online para–social interactions.

## Materials and methods

### Aims

This qualitative study investigated the changing impact of Chinese urban women’s use of the Internet for parenting practices on their daily lives during the COVID-19 pandemic, including changes to parenting practice, parenting psychology, and perceptions of parenting (O’Brien Caughy et al., 2001), and parent-child relationships. Of particular interest were shifts away from traditional parenting practices during the pandemic and how these changes affected participants’ parenting psychology.

### Participants and procedure

The study sample consisted of urban women of childbearing age (mainly mothers born after 1980/1990) from a wide range of industries in China: 47 were born after 1990, 39 after 1980, and 4 after 1970; 49 had a bachelor’s degree, 30 had a postgraduate degree, and 11 had less than a bachelor’s degree. Among the 90 mothers, there were three pregnant women (all of whom received good news about their babies during the study) and one who had a miscarriage in April. The mothers were responsible for 115 babies, including 89 under the age of 6. To protect the privacy of the participants, the names of the participants in the study are presented as numbers (M01–M90; see Table 1).

**TABLE 1:** Participants.

No.	Age range	Education level	Age of children
M01	Post-1990	Bachelor’s	1 year
M02	Post-1990	Bachelor’s	6 months
M03	Post-1990	Bachelor’s	3 months
M04	Post-1980	Bachelor’s	3 years
M05	Post-1980	Bachelor’s	7 years/3 years
M06	Post-1990	Bachelor’s	5 years/2 years
M07	Post-1990	Bachelor’s	5 years
M08	Post-1980	Bachelor’s	7 years
M09	Post-1990	Postgraduate	1 year
M10	Post-1970	Bachelor’s	7 years/6 months
M11	Post-1990	Postgraduate	1 year
M12	Post-1970	Below Undergraduate	8 years/3 years
M13	Post-1980	Bachelor’s	8 years
M14	Post-1980	Postgraduate	5 years/2 years
M15	Post-1980	Below Undergraduate	7 years/1 year
M16	Post-1980	Postgraduate	8 years
M17	Post-1990	Bachelor’s	1 year
M18	Post-1990	Postgraduate	2 years
M19	Post-1990	Postgraduate	4 years
M20	Post-1990	Below Undergraduate	3 years
M21	Post-1990	Bachelor’s	7 months of Pregnancy
M22	Post-1990	Below Undergraduate	5 months of Pregnancy
M23	Post-1980	Bachelor’s	6 years/1 year
M24	Post-1990	Bachelor’s	1 year
M25	Post-1980	Bachelor’s	2 years
M26	Post-1990	Below Undergraduate	3 years/6 months
M27	Post-1990	Below Undergraduate	1 year
M28	Post-1990	Below Undergraduate	Miscarriage in the 4 months of Pregnancy
M29	Post-1980	Postgraduate	5 years
M30	Post-1980	Bachelor’s	6 years/2 years
M31	Post-1980	Postgraduate	7 years/1 year
M32	Post-1980	Bachelor’s	1 year
M33	Post-1980	Bachelor’s	3 years
M34	Post-1990	Bachelor’s	2 years
M35	Post-1990	Postgraduate	3 years
M36	Post-1980	Postgraduate	10 years
M37	Post-1980	Postgraduate	7 years/3 years
M38	Post-1990	Bachelor’s	3 years
M39	Post-1990	Below Undergraduate	6 months
M40	Post-1990	Bachelor’s	5 years/5 years
M41	Post-1980	Bachelor’s	6 years/4 years
M42	Post-1980	Postgraduate	2 years
M43	Post-1990	Bachelor’s	6 years
M44	Post-1980	Bachelor’s	6 years/3 years
M45	Post-1980	Postgraduate	4 years/2 months
M46	Post-1990	Bachelor’s	3 years
M47	Post-1990	Postgraduate	6 months
M48	Post-1990	Postgraduate	2 years
M49	Post-1980	Bachelor’s	2 years
M50	Post-1980	Postgraduate	3 years
M51	Post-1980	Bachelor’s	8 years/3 years
M52	Post-1990	Bachelor’s	4 months of pregnancy
M53	Post-1990	Postgraduate	3 years
M54	Post-1990	Bachelor’s	6 months
M55	Post-1980	Postgraduate	8 years/4 years
M56	Post-1980	Postgraduate	1 year
M57	Post-1990	Bachelor’s	4 months
M58	Post-1980	Postgraduate	6 years/6 years
M59	Post-1970	Postgraduate	2 years
M60	Post-1990	Below Undergraduate	3 years
M61	Post-1990	Postgraduate	2 years
M62	Post-1990	Postgraduate	3 years
M63	Post-1980	Below Undergraduate	7 years/7 months
M64	Post-1980	Bachelor’s	7 years/4 years
M65	Post-1990	Postgraduate	5 years
M66	Post-1990	Bachelor’s	3 years
M67	Post-1980	Bachelor’s	7 years/1 year
M68	Post-1980	Bachelor’s	7 years
M69	Post-1980	Bachelor’s	8 years/3 years
M70	Post-1980	Postgraduate	8 years
M71	Post-1990	Bachelor’s	7 years/2 years
M72	Post-1980	Postgraduate	7 years
M73	Post-1980	Postgraduate	6 years
M74	Post-1990	Bachelor’s	5 years
M75	Post-1990	Bachelor’s	2 years
M76	Post-1990	Below Undergraduate	1 month
M77	Post-1980	Bachelor’s	2 years
M78	Post-1990	Bachelor’s	1 year
M79	Post-1980	Postgraduate	7 years/5 years
M80	Post-1970	Bachelor’s	11 years/3 years
M81	Post-1980	Bachelor’s	11 years/4 years
M82	Post-1990	Bachelor’s	3 years
M83	Post-1990	Bachelor’s	2 years
M84	Post-1980	Postgraduate	7 years
M85	Post-1980	Bachelor’s	6 years
M86	Post-1990	Bachelor’s	3 years
M87	Post-1990	Postgraduate	4 years
M88	Post-1990	Bachelor’s	2 years
M89	Post-1990	Bachelor’s	4 years
M90	Post-1990	Bachelor’s	4 years

Because the researcher is part of the group of urban mothers, the initial participants were drawn from the researcher’s social circle (i.e., friends and colleagues of the researcher). Based on in-depth interviews with these participants, the researcher used the snowball sampling method to obtain a sufficient number of participants to reach 90 participants. Before becoming participants, individuals were informed of the study objectives, volunteered to become study participants, and were given sufficient information to be informed before the start of the interview. After obtaining the participants’ consent, data were collected via Internet phone.

### Instruments and data analysis

This study adopted a mixed-methods design combining semi-structured, in-depth interviews involving both participation and observation. This mixed design method can more objectively obtain data on the experience of Chinese urban women’s parenting practices through mediation during the COVID-19 epidemic. Additionally, the mixed design method can make up for the limitation of data collection of a single research method to a certain extent, and ensure the data collection of the research to the greatest extent can provide solid support for the research findings.

The use of participant observation had two roles in this study: first, to allow the researcher to enter the social life environment of the research subject, participate in the subject’s activities, and gather information through actual personal observation. Ultimately, observation, questioning, feeling, and comprehension can be used to better understand the issues under study. Childcare scenes in daily life can be found everywhere: Communities, parks, shopping malls, playgrounds, hospitals, early childhood centers, supermarkets, and children’s restaurants are public places where urban women with children often gather. These places provide a good opportunity for the researcher to conduct the participatory observation. By observing the daily lifestyles and routines of urban mothers and further participating in their daily parenting activities (holiday gatherings, rituals, and ceremonies), the researcher can maintain continuous contact with them and observe their social interactions in online communities and social media to obtain information about their perceptions and practices in the process of pregnancy, childbirth, parenting, and education. The second role was designed to complement the in-depth interviews and compensate for the shortcomings of the in-depth interviews in the process of data collection. The whole participatory observation ran from December 2019 until June 2021 (i.e., 1 year and 6 months). The observation notes were divided into three parts: handwritten log, electronic memos, and audio recordings.

The semi-structured in-depth interviews lasted approximately half an hour to 1 h, depending on the actual differences between each participant. The purpose of the interviews was to explore the personal and general experiences of urban women during their social interactions and parenting practice through the Internet, especially the changes and differences in their parenting practices and parenting philosophies during the COVID-19 pandemic. To collect more comprehensive and objective data, semi-structured in-depth interviews and participant observation were carried out in parallel, and some of the interview participants were collected during the process of participant observation. We proactively identified urban women engaged in parenting who met our research needs through participatory observation and conduct interviews after observing them for a month. In the interviews, participants shared their parenting philosophies, knowledge, experiences, and changes brought about by their use of the Internet to access parenting information during the COVID-19 pandemic. The semi-structured in-depth interview questions were divided into three categories: personal growth and parenting situation, parenting information access and learning during COVID-19, and perceptions and changes in motherhood during COVID-19 (Table 2).

**TABLE 2:** Semi-structured interview question outline.

Question category	Example problem
Personal growth and parenting	Participant’s profile (age, hometown, education, occupation and income status, among others)
	Participant’s marriage information (marital status, basic information of partner, among others)
	Participant’s mertility information (number and age of children, among others)
	Participant’s parenting information (maternity, care, feeding, consumption, education, among others)
Parenting information access and	Participants’ parenting knowledge gathered from families during COVID-19
knowledge learning during COVID-19	Participants’ parenting knowledge gathered from the media during COVID-19
	Participants’ use and concerns about online parenting during COVID-19
	Information participants acquired before and after COVID-19
Perceptions and changes in	Participants’ perceptions of online parenting knowledge during COVID-19
motherhood during COVID-19	Participants’ perceptions of being a mom and online parenting during COVID-19
	Participants’ perceptions of self-perception, social roles, and online parenting during COVID-19
	Participants’ perceptions of parenting concepts before and after COVID-19

After all interview data and participant observation notes were recorded individually, the text was initially organized and analyzed. The raw data were then analyzed sentence-by-sentence and categorized by thematic keywords using textual analysis to identify specific manifestations of urban mothers’ parenting practices. Different thematic analyses (Brooks et al., 2015) were conducted to identify causal, semantic, similar, structural, and differential relationships among the texts. Due to the specificity of the textual material, some of the texts were analyzed manually and some were analyzed with the help of NVivo. The entire text analysis process took about 6 months to complete.

## Results

The findings suggest that, during the COVID-19 global pandemic, Chinese urban women shifted their parenting practices to the Internet and became actively involved in the mediatized society to overcome the difficulties of parenting practices caused by the obstruction of real communication channels. While aligning their parenting practices with the mediatized society, the psychological changes in their parenting were mainly reflected in three ways: media empowerment, mutual assistance in action, and ideation of motherhood. These changes have also enabled these women to make the psychological shift from traditional experience to scientific parenting and to be more proactive in learning and sharing parenting knowledge, as well as expressing themselves through the Internet. This mediatized parenting practice during the pandemic allowed them to accept the shift in their role as “Internet moms” with a positive mindset in the process of mediatized expression and perception.

### Information empowerment and scientific parenting

During the interviews, we noticed that the convenience of mediatization production (Zhen et al., 2015), information dissemination, and information sharing brought about by the Internet was fully exploited by urban women engaged in child-rearing, who rapidly applied such practices to their child-rearing. It further contributed to their psychological transformation from experienced (or traditional) to scientific parenting. Driven by a common social identity, seeking mutual help through media interaction has become a norm in current motherhood practice. The new generation of mothers—built based on groups for check-ins, mutual support, and experience sharing—is building relationships, sharing experiences, and growing together at the intersection of strong and weak relationships (Granovetter, 1973), releasing the tension between virtual and reality-based media empowerment.

Taking parenting WeChat groups as an example, young mothers’ daily online interactions involve a wide range of information and diverse interaction methods. Topics included motherhood, intergenerational discrepancies (Helga, 2013) in parenting concepts, personal experiences, sharing of parenting knowledge, and mother and baby product recommendations, among others. They also discussed public topics and news events about young children and expressed their negative feelings about motherhood (Lehto, 2020). Information is the basic element of human society, and it forms information chains through different media (Mei et al., 2004), thus providing the underlying support for normal human production and life. When the spatial and temporal boundaries of traditional information dissemination are broken, information begins to diffuse in a non-linear way. In this diffuse flow of information, mediatization has an impact on daily life. The personal lives of urban women raising children were visually presented in this virtual community communication context (Mezgár, 2009). In the interviews, we further found that the media empowerment brought about by the Internet created changes in the psychological aspects of parenting for these women on three levels, including the sharing of goods, memories, and knowledge.

The first level is the sharing of goods. During the interview process, we met a young mother who used to be an overseas parenting product buyer, and there were more than a thousand moms with similar needs on her WeChat. She therefore created a WeChat group called “Parenting Products Unused Exchange Group” and wrote the following on the group’s bulletin board: “Moms who want to sell items can mark up the price and send it to the group, so I can help you share it with other groups. Anyone who likes a certain product can also send me a message.” This WeChat group was very active during the pandemic, and it shared many unused parenting products every day, allowing many young mothers to discuss and interact with each other. The items shared in the group included early learning machines, Lego toys, bubble machines, scooters, excavator toys, intelligent accompanying robots, inflatable swimming pools, children’s pianos, and the like. Receiving more comprehensive parenting information and having more abundant parenting goods are the most important concerns of urban women with children. In traditional Chinese society, parenting goods are obtained through traditional acquaintance networks (Chen et al., 2013), such as family inheritance and sharing among friends. However, during the COVID-19 pandemic, this social sharing bond was cut off. As a result, young mothers tried to help themselves through the Internet. Initially, it was more of an occasional, sporadic behavior based on websites, but later, as this behavior extended to social media, unused parenting goods exchange groups—mainly on WeChat, Sina Microblog (Chinese Twitter), Red Booklet (Chinese Instagram), and Zhihu (Chinese Quora)—quickly became the main place for mothers to find parenting goods. Unlike the sharing of goods in person, the sharing of goods in virtual space proceeds more along the lines of information sharing based on goods. From secondhand transaction in WeChat groups to social media group chats, goods passed between different individuals initially as information, completing a “journey” across time and space. The sharing of goods online is not simply the sharing of goods and information or goods and goods—that is, the sharing of these objects carries not only the physical objects themselves but also the emotional connection with urban women raising children based on the sharing of objects, combining motherhood and practice in a material way (Turner and Norwood, 2013). This media-based sharing of goods both mediatizes the parenting practices of these women and even involves the goods in this mediatization process. The concept of scientific parenting is also spread through the “physical-emotional” interaction field (Lewin and Cartwright, 1951) formed by the interaction between people and goods and between people and people, promoting deep acceptance of this concept.

The second level is the sharing of memories. During the participatory observation process, we discovered an application (app) called Qinbaobao for which young mothers form the main user group. Users can upload photos, videos, and parenting diaries, and the uploaded information is intelligently categorized according to date and content, making it easy to find. After posting a message, the user can invite her friends and family members to join her parenting group; they can browse, comment, like, and share the message after entering. Examples include “Baby’s first month of life story” or “How to be a good mother while working” and other stories about the growth of young children (Johnston and Swanson, 2006). The app also has an interactive community function to meet users’ needs for online communication and information sharing. As a digital form of self-representation (Blum-Ross and Livingstone, 2017), users can share snippets of their parenting lives through the app, and other users can comment on, like, and share those snippets. Some of the app community leaders have reached 100,000 followers because they often share photos and content in the community. In the traditional Chinese parenting model, photos or images of the first month after birth, birthdays, and school promotions are special, precious parenting memories. However, as mediatization continues to permeate human daily life, this traditional pattern of only special nodes being remembered is gradually breaking down. This media-driven de-nodalized mode of memory has further changed mothers’ memories of parenting during the COVID-19 pandemic, as well as their attitudes toward parenting memories. During the COVID-19 pandemic, urban mothers not only recorded and shared their parenting memories, but also found new spaces for self-expression and self-fulfillment, and found psychological reliance on the group via media-based community interaction (Thorns and Eryilmaz, 2014). With the mediatized connection, these women seek emotional comfort by sharing their parenting stories, helping each other by sharing parenting information, and seeking value recognition by sharing the joint growth of parents and children. If sharing in traditional societies focused more on the entry point of the individuals who practice motherhood, the sharing of women’s motherhood memories online tends to focus on the environmental and cultural conditions of motherhood performance (Enzhi, 2019). The formation of a sense of belonging and identity in the collective memory of media-based community interactions is oriented toward shaping the psychological value of parenting for mothers—more so than traditional parenting models—and can lead to a positive shift from a “child-centered” (Saldinger et al., 2004) to a “mother and child-centered” approach in the psychology of parenting for urban child-rearing women.

The third level is knowledge sharing. Through various media platforms, young mothers have more access to parenting knowledge, and in the process of communicating with others with similar parenting experiences, new parenting knowledge takes shape as they learn from and communicate with each other.

*Constipation during the COVID-19 pandemic was a headache for mothers, and I had no experience in dealing with this problem myself, nor did I have any knowledge of parenting. Fortunately, there was a mother in my WeChat group who had the same problem with her child. She gave me a lot of advice and recommended probiotic products. I myself also learn about probiotics on mother and baby forums*… *(Excerpts from M62 interview materials)*

In various WeChat groups and parenting apps, parenting knowledge is fragmented across the network; this information is enriched, supplemented, borrowed, used, questioned, and absorbed in the exchange among mothers. Chinese society has undergone a profound transformation since the 1980s and, in the process, moved from tradition to modernization. The highly compressed modernization process has had a great impact on ideas about parenting (Meng, 2020), and much parenting knowledge has been abandoned in the wave of progress. In the interviews, we found that many young mothers born after 1980/1990 questioned traditional parenting knowledge. In the parenting knowledge system of older generations, there are no terms such as probiotics and Heimlich method. However, the new generation of mothers who have been nurtured by online parenting knowledge can respond to these professional terms proficiently. “Probiotics can promote the absorption of children’s gastrointestinal function,” “If the food is stuck, the Heimlich method needs to be used immediately”… They have taken the initiative to shift the channel of learning about parenting from home to society at large and from in-person interactions to the Internet, ultimately coming to use the media as their main source of parenting knowledge. This kind of learning was initially only available to some mothers. With the outbreak of the COVID-19 pandemic, this mode of learning has rapidly become popular among urban women raising children, given the huge demand for parenting knowledge. What urban women learned about parenting through the media during the COVID-19 pandemic not only made up for their own parenting shortcomings, but also gave them solid support for their beliefs in scientific parenting, which helped them to psychologically accept, agree with, and support the concept. This psychological change through mediatized parenting practices is continuing beyond the COVID-19 pandemic and has extended from the virtual to the real. It is having a concrete impact on the real lives of urban mothers. This impact is reflected in their lifestyles, consumption patterns, and many other dimensions, and it is likely to be passed on to their children.

### Mutual action and parenting community

In a mediatized society, the media exerts a constant influence on daily life as an internal driving force. Although media interactions superficially bring about changes in the perceptions and values of urban women, ultimately the psychological changes brought about by these media interactions are also applied to social practices through their parenting. In the interviews, we found that, during the COVID-19 pandemic, the online community generated effective media mobilization through a strong sense of collective emotion and solidarity, whether it was through the mutual information support of the WeChat group or the social interaction of social media. The media mobilization would also further lead urban mothers to form a collaborative, expressive, and active parenting community. The mothers support each other psychologically, help each other in action, and express their opinions, which is a positive step for urban women in defending their rights and interests and participating in social topics.

The first aspect that needs to be considered is the collaborative community. During the COVID-19 pandemic, mothers exchanged goods and shared experiences through the Internet over a long period. Mothers gradually shared similar values and views on parenting and also built a foundation of trust, which was a prerequisite for the creation of a collaborative community. In our interviews, we found that urban mothers used WeChat groups and other social media not only to share their experiences but also to watch over each other and co-parent. During the interviews, we met a mother (M15) from Beijing who was invited by her sister to join a group called “English, Chinese, and Math Parents’ Group” during the pandemic, with 87 mothers in total. Her sister told her that she should follow everyone in the group to learn about parenting, to see what children are learning now, what is being talked about in the field of early education, and to learn more about schooling, which would be useful in a couple of years.

*After being invited into the group I found that there was a lot to learn. The group is set tasks every day, and the mothers monitor each other. Everyone is willing to participate. I found that it made quite a difference to both my children and me…*

*When mothers in the group share their children’s growth process or achievements, I will always unconsciously compare them with my own children, or use them as a reference. This strengthened my confidence in sticking to interactive learning with other mothers in the group…*

*(Excerpts from M15 interview materials)*

Through the WeChat group, mothers could share photos or videos of their children’s learning in the group every day, which triggered other members to imitate them and create a scene of common learning and sharing in the group. Although the sharing of lessons learned is an important element, this practice is also a cyber-ritual for mothers to collaborate (Jacobs, 2007). On the one hand, it is a self-presentation from multiple narrative subjects, and on the other hand, it co-constructs a mediatized parenting discourse, providing concrete and intuitive reference perspectives for other mothers. There are also WeChat groups for pregnant women, which have been set up to provide help to urban mothers during the pandemic. One such group has no less than 500 chat messages per day, covering topics such as fetal movement communication, maternity confusion, pregnancy anxiety, vaccination exemption (Tangherlini et al., 2016), and the spread of the COVID-19 pandemic. These messages are updated quickly and frequently interact. People also share experiences and resources in the group to provide practical offline assistance: “My baby was born at 7 pounds, and it was still a normal birth, it was so painful to give birth,” “I had a cesarean 2 days ago, and my wound is still painful,” and “Don’t be afraid, we are with you.” Through sharing their experiences in preparing for pregnancy and daily life, they encouraged each other, supported each other, built confidence, relieved anxiety, brought each other great psychological support and assistance with practical information, and tided over the difficulties together. Some of the WeChat groups also have volunteers who make manuals for pregnant women to protect themselves from the pandemic and await their delivery with peace of mind or provide attentive company to lonely and helpless pregnant mothers, answering their questions at any time and relieving tension and anxiety. Through this mutual support, the volunteers communicate with pregnant mothers promptly and help them with maternity checkups, hospital appointments, contacting family members, purchasing daily necessities, and other matters. Although this collaborative action is transmitted in the form of media, it is real for both the individuals who transmit the information and for those individuals who act on it. Thus, the media-based collaborative community not only influences the parenting practices of urban women but also contributes to their psychological formation of an individualized collectiveness (Soon and Kluver, 2014).

The second aspect is the expressive community. During the participant observation, we found that WeChat groups were most likely to generate retweets from mothers on topics such as food safety and defective toys. The voices of a single individual are not enough to attract widespread attention, so in this case, seeking collective voices online is a relatively convenient and feasible path. Mothers gather together through online communities, driven by the same or similar social identities and social experiences, to transform individual expression into collective expression, thus enhancing the right to speak out and express themselves at events, and achieving social resistance and balance.

Pregnant mothers in Wuhan became a special group during the COVID-19 pandemic. At the beginning of the pandemic, major hospitals focused attention on treating COVID-19 patients, and even specialized maternity and children’s hospitals were included in the pandemic treatment system. It became difficult for pregnant women in Wuhan to find either standard maternity appointments or hospitalization for delivery. In the absence of help from society at large, pregnant mothers began to express their voices online and on social media, hoping to spark societal attention. The dense network of information garnered the interest of web celebrities and news media, and this “neglected group” quickly gained attention through their intervention. On January 26, 2020, the “Wuhan Pregnant Women WeChat Group” was established to help pregnant mothers in Wuhan during the COVID-19 pandemic; the following message was posted on the WeChat group’s web bulletin board: “We provide medical information, vehicle information and psychological counseling.” At the same time, there was also the “Hubei Maternity Free Consultation Group” organized by local doctors in Wuhan, with more than a dozen professional doctors from all over the country promptly replying to consultations and requests for help from group members. Urban women raising children through the media quickly gathered many voices, to express their needs, not only to convey their voices to the community, and receive the support of society. The key factor that enables the formation of media communities is that they transcend existing social relationships and give them new life by bringing people with diverse life backgrounds and social experiences together. This further promotes the psychological homogenization of cognitive and behavioral patterns in the process of interaction and communication. Urban parenting women make the most of social interactions at the mediated level, trying to break out of the constraints imposed on them by family life at the spatial and temporal level. Through more frequent and closer contact with others and society, whether close by or distant, they can improve their relationships and lifestyles, thus embracing a wider range of living spaces. The community of expression formed by urban child-rearing women during the COVID-19 pandemic, although superficially for self-help, behind the scenes shows a profound awareness of mediatized expression and a more psychological concern for the self on the part of these women. They are trying to make their voices heard, and these voices are not only making society more aware of them as a group; but in doing so, the group itself becomes more psychologically aware of its own power and the power of mediatized society in their daily lives. The psychological shift in parenting topics from private to public triggered by media expression during the COVID-19 pandemic is likely to affect more and more urban mothers, who could continue to actively express their voices through the Internet media to defend their rights and interests and to participate in social and public issues.

The third aspect is the active community. During the interviews, we met a mother from Hangzhou (M73) who shared a process that appeared during the COVID-19 pandemic in which mothers communicated with the kindergarten through media interactions; this mother showed us the chat logs in the WeChat group. Her daughter attended a small, private kindergarten with a high fee, and the school infrastructure was satisfactory. The school advertises a Canadian education system and faculty, with daily classes with foreign teachers. Parents have a classroom WeChat group for communication and a “Baby’s Home” group created by a dozen mothers. One month before the summer holiday, the class visual education teacher was reassigned because the school was opening a new campus, and the parents of the students were not informed. The moms started discussing this issue with each other in the “Baby’s Home” WeChat group.

*Parent A: “Hello everyone, I heard that Shiny, the school’s visual arts teacher, was reassigned and the school did not inform parents in advance. It seems that Annie, the dance teacher, has also resigned. These two teachers are very popular with the children, so let’s talk about it and see if we can talk to the school.”*

*Parent B: “My son said he hadn’t been to visual arts class for many days, and he was crying in the morning.”*

*Parent C: “It’s not good for children to change teachers so often.”*

*Parent D: “Well, I think we have to unite to get the school’s attention.”*

*Parent A: “Let’s go together tomorrow and ask for a tuition refund. It’s too disrespectful to children and parents.”*

*Parent C: “The key is that the tuition is still so high and the teachers are still unstable. If the school cannot deliver on the promises made prior to enrollment, we should be given a refund.”*

*Parent B: “Many of the classes have not lived up to the previous promise. The very beginning promised to have drama class, and I haven’t seen it. The school promises to give students daily English communication before enrollment, and I feel that there are very few opportunities for this on a regular class*…”

*(Excerpt from M73’s WeChat group chat log)*

The WeChat group went from initiating messages to exchanging messages, negotiating ideas, initiating actions, and ultimately organizing actions in just half an hour. The discussion among the mothers gradually spread from the initial teacher reassignment to recent teaching content, the flashy curriculum, excessive tuition, food safety, undersized playgrounds, substandard educational commitments, and a host of other issues, which mothers normally tended to ignore or felt the school would not pay attention to were listed in this chance in-depth discussion. The mothers started soliciting issues and agreed on a specific time to go face to face with the school representatives. According to a follow-up from the mother (M73) on how things were going, the mothers discussed and decided that four parents would go as representatives to communicate with the school, and some of these issues were later resolved. For women, integrating into a larger group through media communities and forming a “community” through daily communication and interaction can not only reduce the individual’s ability to overcome risks in the face of uncertainties but can also create a link between discourse and action, from offline to online, driven by similar life experiences, giving rise to virtual and real action practice (Miller and Madianou, 2012). This WeChat group was not only a kind of mutual help in action but also a kind of psychological mutual help, which made a positive contribution to further strengthen the identity of the child-rearing community.

During the COVID-19 pandemic, urban women’s media action not only reduced their stress and anxiety in the face of external events but also increased their confidence in speech and action through collective action. This media action support allowed urban mothers to come out from the limits of individual experience and feel psychologically that they are not just independent individuals, but that they have something to rely on, they have support, which enhanced their sense of well-being (McDaniel et al., 2012). The COVID-19 pandemic may eventually end, but the impact of the parenting community formed by online mutual support on the psychology of urban mothers may not. In the future, the online and offline parenting model could play an increasingly important role in the parenting practices of urban women, further stimulating a sense of autonomy in action and encouraging more group members to team up for active sharing and voluntary support.

### Motherhood imagination and media shaping

During the COVID-19 pandemic, a wide variety of news and information on “how to be a good mother” was published, and the images of “super mothers” and “perfect mothers” in dramas and variety shows were completely different from traditional mothers. The new view of parenting shaped by the media has influenced urban women’s psychology and practice of parenting.

For urban mothers, digital technology and social media are gradually shifting forward in time to the moment when they feel they “become mothers” and use their power to construct the image of motherhood imagination among mothers. In the interviews, we often asked, “When did you feel that you had become a mother?” The responses we heard at the beginning of the COVID-19 pandemic tended to be: *“When the baby cried loudly” (M04)* or *“When the baby in my womb kicked me during a pregnancy test” (M42).* This question may have a new answer given the media interactions of urban mothers during the COVID-19 pandemic, as many women are already learning online about parenting while pregnant. Mediatized parenting is gradually becoming a new option for more and more urban women to interact with their children during the pandemic. During the participatory observation, we found an app called Babytree, which is divided into four sections: parenting, social interaction, shopping, and pregnancy services. The app provides pregnant women with information about recipes, fetal education, maternity check-up, and fetal movement in different stages of pregnancy, and can also answer questions from pregnant women.

*“When I was first pregnant, I used to browse Babytree, which was so informative that I didn’t have to buy any parenting books.” (Excerpts from M19 interview)*

*“Every time I go for a maternity check-up, I can’t say a few words to the doctor, and I can’t read the lab reports I get back. So, the information about exactly is the condition of one’s body, other than the bulging tummy visible to the naked eye, is almost unavailable. But through the media, it is possible to see the basic condition of the child at each stage of pregnancy.” (Excerpts from M90 interview materials)*

The app also offers pregnant women and new mothers a variety of social networking options to share news of their pregnancy with other pregnant women. This is unimaginable in traditional Chinese societal norms for pregnancy and parenting practices. According to traditional Chinese customs, pregnant women are not allowed to share the news of their pregnancy with others during the first 3 months of pregnancy. Now, however, pregnant women can connect and share their experiences with other mothers in pregnancy preparation or preparing to become mothers on the Internet. It has been both a joy to share and an enriching experience for my own pregnancy preparation.

We also found that, during the COVID-19 pandemic, many mothers chose to announce their motherhood by displaying ultrasound images of their fetuses on social media. The media not only creates a physical attachment between mother and baby but also builds on it again to create a closer parent–child interaction. The different apps cover almost all maternity and parenting content for women before, during, and after they become mothers. The figures, 3D animations, and anthropomorphic words used in the app to present the indicators of the child’s growth and development are all attempts to create a warm parent-child bond, to help mothers construct a concrete image of the baby inside the womb and increase intimacy. At the same time, the data records of the pregnant women in the app, the maternity reports, and the nutritional advice sent to pregnant women are constantly reinforcing the role of pregnant women as mothers and the motherly responsibilities they should take up for the health of their fetus. Media thus shapes responsibility and achieves the construction of intimacy. From pregnancy to a fetus, from unfamiliarity to familiarity, and from maternity checkups to nutrition, urban women gradually start their role initiation as mothers while using the media.

During the participatory observation, we found that the presentation of parenting in the media was not only a presentation and discussion of the current reality of parenting in China but also a reconstruction of this central topic in life through narrative. Through this format, the online media attempts to reproduce the contradictions and conflicts that arise in the process of parenting, so that mothers can empathize and be inspired, as well as seek to guide value choices and parenting concepts that are in line with social development. In the concept of traditional Chinese society, a mother must completely give up her ideals, goals, and even her own life to provide her children unreserved love and unconditional care, and to meet the society’s image of “superhuman” and “perfect” mothers and the expectation of “anything can be done.” However, as the American poet Adrian Rich said, “If the role of women is compressed to almost the same time as the role of mother, the value of the female individual is eliminated.” In the past 2 years, with the media’s extensive discussion of parenting topics, more and more urban women of childbearing age have begun to rethink about their own life situations and reflect on their roles. Some women also took to social media with the slogan “I’m mom, I’m myself.”

*“Don’t just praise the greatness of mothers, every mother is a living person, affirm the value of mothers, see the dedication of mothers, whether she is a housewife or a professional woman, whether she is beautiful or aging. It is not natural for mothers to give to their children, we need to understand the selfishness of mothers and be less demanding of them.” (From the social media account of the network name “Mengmeng,” the excerpts have obtained her consent).*

The media’s central reflection of social values and consciousness has also become an important force in the formation and development of new social concepts. During the COVID-19 pandemic, diverse media formats—whether it was news and information that communicated scientific parenting concepts, or films and television productions that portrayed diverse images of mothers—have been providing a media mirror of current Chinese parenting issues. These imaginings of reality, in turn, continued to influence urban mothers’ ideas about and practices of motherhood.

In China, dramas are characterized by high social acceptance and strong penetration. During the COVID-19 pandemic, mothers were not able to participate in normal social interactions, so the purpose of watching films and dramas for leisure and entertainment via online media platforms became one of their main ways to relax. Influenced by social identity and life experience, movie and TV dramas with child-rearing themes are most popular among urban women with children. The study found that during the pandemic, the image of mothers in parenting dramas was no longer the traditional one-size-fits-all, but included various types of maternal roles, including working mothers, single mothers, and mothers of second children. These rich maternal roles are deeply rooted in the hearts of people through the popularity of dramas, and many women, while watching the dramas, also empathize with the power of the characters in similar life situations and see their reflections in the characters. In the participant observation, we found that the very contrasting maternal roles that appeared in the film and TV series *A Love for Dilemma*, which aired in 2021, were welcomed by the mothers. The two mothers, one who lets her children develop on their own and the other who cares for them in every way, map out their different parenting philosophies in the real world. These contrasting models of motherhood truly restored the diversity of mothers in real life, presenting the different parent–child relationships and ways of getting along in different families in daily life. In *A Love for Dilemma*, a mother who had previously left her child to develop on her own is confronted with tremendous parenting anxiety, resulting in a psychological change leading to the question, *“Am I a bad mother? [*…*] I should have started paying attention to her learning long ago!”* Self-doubt and self-denial sent her into deep anxiety about her parenting style.

Some movies and dramas feature a range of motherhood models, from the stay-at-home-mom who is weak and subservient to her husband to the working mom who is brave enough to leave her family and start a successful business. This transformation of maternity presented through the media is extremely touching for urban women in similar life situations who are raising children. During the COVID-19 pandemic, urban mothers have also actively compared the images of mothers presented in the media with themselves, and have tried to change their parenting psychology and deepen their role identity. Through social media and WeChat groups, urban women express their views and perceptions of the images of mothers portrayed in dramas in comparison to their own lives. Through constant interaction with the media, these mothers are more likely to gain group recognition, thus contributing to the awakening of urban women’s self-awareness. This change is not only helping urban women overcome the misconceptions of traditional parenting and form a scientific parenting mentality but also subconsciously encouraging women to break through themselves, strengthen their perception of self-presence (Daniel and Mirca, 2012), get out of the family, and realize their own value in life.

Parenting shows are also a medium of expression that has received attention from urban women raising children. Unlike the idealized mothers in traditional media, these shows present the experience of motherhood and parenting through mundane, everyday details (Lopez, 2009). During the pandemic, parenting shows simulated the lives of different families, showing the conflicts and contradictions that may arise during the parenting process, as well as the diverse parenting approaches in different families. In the participant observation, we found that, at the beginning of the COVID-19 pandemic in 2020, more than ten parenting shows were launched in China. Some of these shows were child development-centered, while others were focused on mother-parenting, education sharing, or the family relationship. Each type of show delivers different parenting psychologies and practices to mothers. In the participant observation, we found a show called *Mom Is Superman*, which enhances the emotional development between parents and children through parent-child interaction in which the mother and child participate in activities together. This promotes the growth of both mother and child. Parenting shows are a reflection of parenting anxiety and women’s demand for autonomy in the context of the COVID-19 pandemic, as well as the social construction of the connotations of motherhood. Through documentary-style observation, variety shows have set up perfect mother role models, and these have influenced the identity of urban women raising children. It is worth noting that during the pandemic, watching parenting variety shows has prompted mothers to think about the psychology of parenting, their role in their children’s education and upbringing, and the reality that fathers are not particularly involved in the parenting process in many families in China today. These reflections were accompanied by online media interactions that quickly gained the participation and discussion of other mothers with a broad social consensus on parenting. At the same time, these reflections may lead to a change in the concept of childcare for urban women, and hopefully, raise the awareness of “motherhood” and “womanhood” in society, ultimately leading to more attention and support for women.

## Conclusion

This study sought to identify issues connected to the relationship between online behaviors and the psychology and practice of parenting among urban mothers in China during the COVID-19 pandemic. We found that COVID-19 undeniably caused many inconveniences for urban women with children; however, these women were also found to be actively using the Internet to gain knowledge and help each other. The active integration with the mediatized society created new opportunities for this group. Through the analysis of empirical data obtained from in-depth interviews and participant observation, we found that the psychological changes triggered by Chinese urban women’s mediatized parenting practices during the COVID-19 pandemic primarily appeared in three aspects: changes in the concept of scientific parenting due to information empowerment, identification of a parenting community due to mutual assistance in action, and motherhood imaginaries shaped by the media. From experienced parenting to scientific parenting, from family to society, urban mothers were observed to be interacting with each other on the Internet about parenting knowledge, parenting psychology, and social relationships. These changing influences complemented and reinforced each other and together influenced urban women’s psychology of child-rearing and awakened their concern for their social rights and participation in social public issues in a mediatized society.

## Discussion

This study examined the parenting practices in which urban Chinese women with children engaged online during the COVID-19 pandemic, as well as the changes in the psychology of parenting that resulted from these practices. In an evolving mediated society, the use of the Internet for social interaction is a part of normal social activities. However, before the outbreak of the pandemic, urban women engaged in online parenting practices that were fragmented and isolated, complementing real-world social parenting practices. At the beginning of the pandemic, many mothers also tried to reconstruct the structured life they had before (Kusin and Choo, 2021). However, as health prevention policies became more restrictive, urban mothers began to try to move away from traditional social patriarchal institutionalization and experiment with purposeful maternal childcare practices via the Internet (Green and Joy, 2015). As more and more mothers joined, the Internet gradually became the main way for urban women to acquire parenting knowledge, share parenting experiences, and engage in social interactions during the pandemic. This virtual practice also influenced, to a certain extent, urban women’s psychology of parenting, and these psychological changes appeared in their online participation, transition in parenting identity (Richter et al., 2021), identification with the parenting community, and imaginaries of motherhood.

Importantly, these women have become psychologically free from the shackles of traditional Chinese social parenting customs and concepts, and have formed an ideal discourse community of scientific parenting supported by the interactive online parenting community (Mannay et al., 2018). Due to shifts in traditional interpersonal communication boundaries through online sharing (Davide and Laura, 2021), urban women with children can break through the acquaintance circle and go beyond the family circle to find groups with similar life experiences online. These women face similar problems, have similar experiences, and are more likely to receive psychological support; they have similar social backgrounds, have similar knowledge and education, believe in science, and are more likely to accept the concept of scientific parenting. During the interviews, we found that these mothers generally felt that the COVID-19 pandemic period was a new window for them to get to know themselves and gain knowledge about parenting. They saw the outside world through the Internet, made new friends, and noticed problems in their daily parenting process. This has created a positive meaning for them and allowed them to accept the concept of scientific parenting more actively and change from family to scientific parenting. Some mothers believe that the traditional view of childcare formed over China’s long historical development is still deeply rooted and that many customs and experiences create physical and psychological restraints on women who are raising children. Although there is a growing awareness that parenting has a different meaning for women (Bermúdez et al., 2014), there still has not been much attention paid to this group of women and childcare. During the pandemic, urban women’s online parenting practices helped them to break free from the traditional customs of Chinese society and form a space for sound scientific parenting. At the same time, the parenting community formed by online practices has enabled urban women to engage in the process of meaning-making for parenting practices (Siu-ming et al., 2021), helping them to relieve mental stress, seek psychological comfort, and obtain a safe harbor of group support, which is conducive to the group’s ability to unite and express their voices.

Our study also noted that, during the pandemic, urban mothers improved their ability to deal with childcare issues through online practices and gained a dawning of female autonomy (Fierloos et al., 2022). Even so, we need to acknowledge the limited existence of parenting topics as social mirrors on the Internet. Fertility issues present a large number of diverse types depending on individuals, families, regional cultures, ethnic religions, and many other factors. Online parenting practices during the pandemic also created realities that cannot be ignored: the risk of child abuse due to online stress (Chung et al., 2020; Yamaoka et al., 2021), parenting mental health (Kurata et al., 2021; Marzilli et al., 2021), and the stress of parenting due to the epidemic should all be given attention (Ben-Ari et al., 2021; McRae et al., 2021). Although these problems are particularly prominent due to the parenting practices of urban women raising children during the COVID-19 period, they have also profoundly affected urban women’s understanding of parenting topics. However, for Chinese society, there are deep-seated social factors in these impacts. Continued sluggish fertility rates, rapidly aging populations, high childcare costs inhibiting the willingness to bear children, and the widening gap in parenting awareness all affect urban women’s views on parenting to a certain extent. It is difficult to link media production, circulation, and reception to such a wide range of intersecting social and cultural arenas based on simple network participation. It is even more difficult to fully understand the social impact and cultural significance of the network in the daily lives of our research subjects. The government, media, and social organizations should therefore pay more attention to the group of urban women raising children, focus on protecting women’s rights and interests, solve the problems they encounter, and make them feel secure in their role in society. At the same time, these mothers should be guided to actively participate in offline social interaction activities through parenting seminars and psychological counseling.

Currently, many countries around the world are still coping with the COVID-19 pandemic, and women in these countries are experiencing similar problems to those in China. We were limited in our ability to obtain information on women’s experiences with online parenting practices during the COVID-19 pandemic in other countries, which is a limitation of this study. Future studies could be expanded to more countries, involving more participants, and obtaining richer empirical information. This research also only focused on the influence of online communities on urban mothers’ psychology of parenting and did not consider factors such as fathers, children, and family type. Parenting is an all-encompassing social issue. Future research should pay attention to collecting empirical data from the perspective of other members of the family, as well as comparing urban mothers with other family members. This could help to further identify the psychological and operational changes in parenting brought about by online parenting practices; moreover, positive implications could also be achieved for the promotion of scientific parenting concepts.

## Data availability statement

The raw data supporting the conclusions of this article will be made available by the authors, without undue reservation.

## Ethics statement

Ethical review and approval was not required for the study on human participants in accordance with the local legislation and institutional requirements. Written informed consent from the patients/participants or patients/participants legal guardian/next of kin was not required to participate in this study in accordance with the national legislation and the institutional requirements.

## Author contributions

RZ: investigation, formal analysis, and writing – original draft. GJ: conceptualization, investigation, and writing – review and editing. Both authors contributed to the article and approved the submitted version.
